# The water relations and xylem attributes of albino redwood shoots (*Sequioa sempervirens (*D. Don.) Endl.)

**DOI:** 10.1371/journal.pone.0191836

**Published:** 2018-03-28

**Authors:** Jarmila Pittermann, Joshua Cowan, Nathan Kaufman, Alex Baer, Elaine Zhang, David Kuty

**Affiliations:** 1 Department of Ecology and Evolutionary Biology, University of California, United States of America; 2 Urban Adamah, Berkeley, California, United States of America; 3 Department of Biology, California State University, California, United States of America; 4 College of Arts and Science, University of San Francisco, San Francisco, California, United States of America; 5 Henry Cowell Redwoods State Park, Felton, California, United States of America; Estacion Experimental del Zaidin, SPAIN

## Abstract

Plants that lack chlorophyll are rare and typically restricted to holoparasites that obtain their carbon, water and mineral resources from a host plant. Although not parasites in the traditional sense, albino foliage, such as the sprouts that sometimes develop from redwood tree trunks, are comparable in function. They occur sporadically, and can reach the size of shrubs and in rare cases, trees. Albino redwoods are interesting because in addition to their reduced carbon resources, the absence of chloroplasts may impede proper stomatal function, and both aspects may have upstream consequences on water transport and xylem quality. We examined the water relations, water transport and xylem anatomical attributes of albino redwoods and show that similar to achlorophyllous and parasitic plants, albino redwoods have notably higher stomatal conductance than green sprouts. Given that stem xylem tracheid size as well as water transport efficiency are nearly equivalent in both albino and green individuals, we attribute the increased leaf water loss in albino sprouts to lower leaf to xylem area ratios, which favour improved hydration relative to green sprouts. The stems of albino redwoods were more vulnerable to drought-induced embolism than green stems, and this was consistent with the albino's weaker tracheids, as characterized by wall thickness to lumen diameter measures. Our results are both complementary and consistent with previous research on achlorophyllous plants, and suggest that the loss of stomatal control and photosynthetic capacity results in substantial vascular and anatomical adjustments.

## Introduction

The vast majority of plants are photosynthetic autotrophs, so encounters with achlorophyllous plants are uncommon and typically constrained to parasites. For example, a number of holoparasites such as *Monotropa* and *Rafflesia* spp. are entirely devoid of chlorophyll, relying instead on their host for sugars and nutrients, while variably chlorophyllous plants such as the rare orchid, *Cephalanthera damasonium*, compensate for their condition by forming strong associations with mycorhizzae [1]. In each example, the pigment-less plants are under strong selection to maximize fitness by association with another species, that is a host. However, rarer still are persistently achlorophyllous plants for which the absence of chloroplasts is the result of some genetic or developmental instability rather than an evolved life-history strategy. Such ‘albino’ vegetation is a sink for water and carbon, so its survival is entirely dependent on ready access to these resources. In fact, if the albino is attached to a photosynthesizing mother plant, it can survive indefinitely, often for decades. Such is the case with redwood albino (*Sequoia sempervirens* (D. Don) Endl., Cupressaceae) sprouts, which although scarce, are persistent entities throughout California’s redwood forest range.

**Fig 1:**
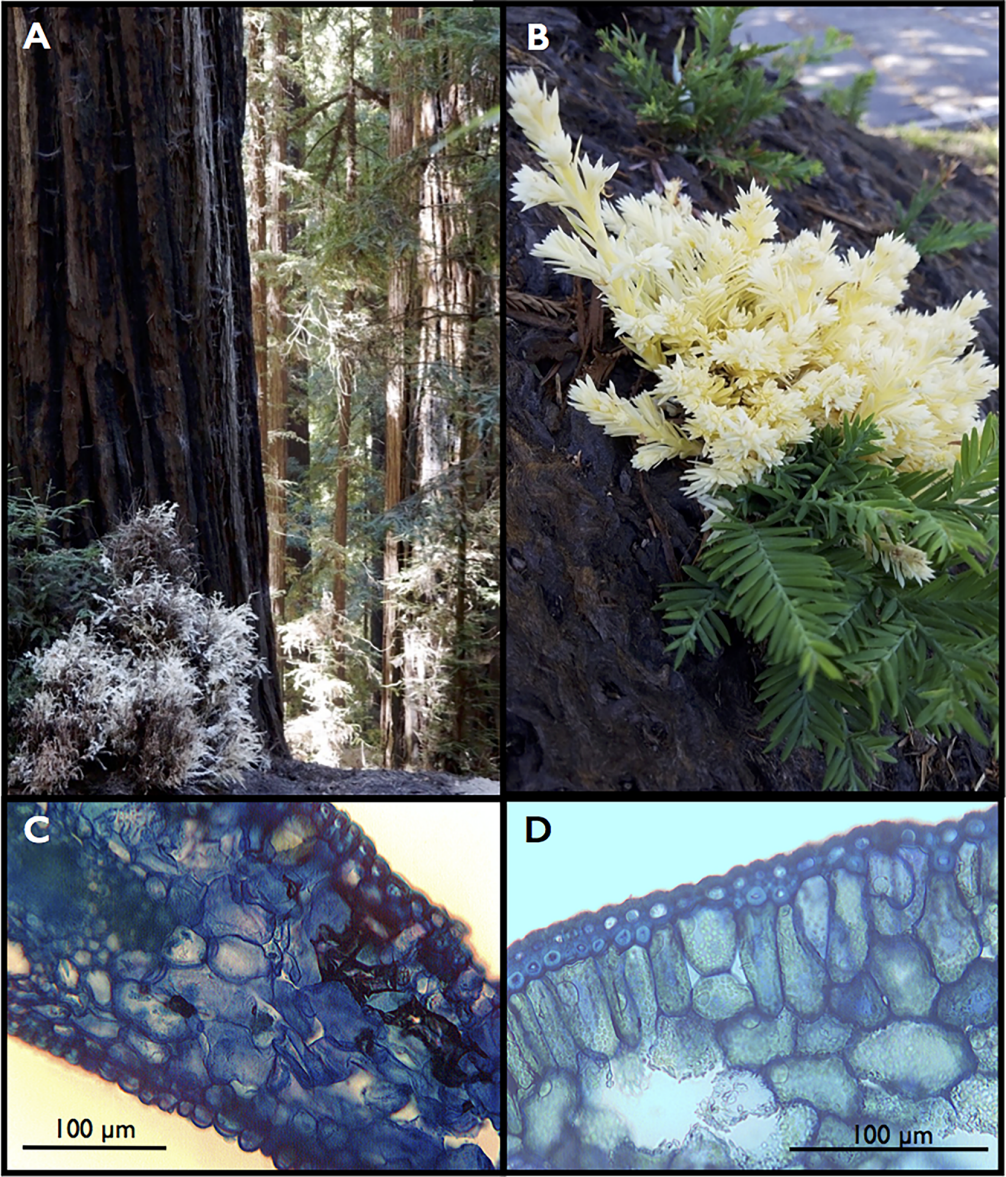
Albino foliage *in situ* and in cross-section. The albino shrub in panel A is about 1m in height and growing from a burl at the base of a large redwood tree. Green sprouts emerge from the left side of the burl. Albino foliage typically develops more slowly than adjacent green foliage (B; the green phyllode is ~7cm in length). Transverse section of an individual albino leaf (needle) reveals no chloroplasts and more tightly packed mesophyll (C), while many plastids are visible in the green leaf (D). (Photo credits: A, D. Kuty; B, Jessica Friedman; C and D, A. Baer).

Albino sprouts are examples of long-lived achlorophyllous vegetation. Arising directly from the tree trunk or from a burl, they resemble their green counterparts in nearly every way, except that their sparse leaves may be chimeras, that is only partially green, or the leaves may be entirely lacking in pigment, and strikingly white (Fig 1A and 1B).

They are also softer to the touch, with a less developed cuticle and a rubbery texture that is unlike the surface of normal sclerophyllous redwood leaves. The woody stem of albino sprouts may be slightly paler in colour, but once mature, the appearance of the bark is otherwise similar to equivalently-sized green sprouts.

Observed across the redwood range, albino sprouts are generally small and inconspicuous, but in a few instances, they may reach the size of shrubs. Some albino redwoods, such as the Cotati redwood in California’s Sonoma County, are impressive in their extent: albino or chimeric foliage comprises nearly half of this bushy, 12 m tall tree, with some branches even producing cones [2]. Over 40 unique sightings of albino redwoods have been documented in Santa Cruz county alone; each specimen is at least one shoot but could be many more. The number of albino redwoods may actually be under-reported because the sprouts are often ephemeral. They appear to be more summer deciduous than green sprouts, perhaps in response to drought, while some twigs die back over the wet winter season probably due to the reduced photosynthetic activity of the parent plant.

The physiology of albino plants is interesting because in addition to lower carbon resources, proper stomatal function may be disrupted in the absence of chloroplasts, and shifts in both traits may have 'upstream' consequences on various aspects of water transport. This was in fact observed in the leaves of albino *Citrus* and other angiosperms, which had significantly greater transpiration rates, higher stomatal density and a slightly higher fraction of large-diameter vessels in their petioles than their green counterparts [3,4]. *Citrus* plants are susceptible to mutations because repeated grafting leads to genetic instability, while albino mutations in redwoods are a natural phenomenon. We wondered how the water relations and vascular attributes of albino redwood sprouts differ from their green counterparts, and if the response is comparable to what has been reported in albino angiosperms. We endeavoured to answer these questions by studying the water relations, hydraulic function and xylem structure in vigorous redwood albino sprouts. Our results are both complementary and consistent with previous research on albino angiosperms and holoparasites, and suggest that the loss of stomatal control results in a common upstream vascular response, even in distantly related species.

## Methods

### Plant material

Albino redwood growth is typically small and inconspicuous with only a few woody shoots, but we identified four individuals with woody stems that were sufficiently comparable in size to adjacent green-leaved material. Two such shrubs exist in Henry Cowell State Park in Felton, California; one densely woody shrub emerges from a ground-level burl of an approximately 1.5 m diameter tree (Fig 1A), while another shrub grows from the decayed remains of a harvested or toppled redwood. These plants are protected and sampling is not typically permitted. The other two albino individuals are located at the Fernwood Resort, a campground in Big Sur near the southernmost extent of California’s redwood forest, where they form prominent shrubs emerging from old tree stumps. Albino phyllodes are markedly more deciduous than those of green trees, so short sprouts and heaps of dead material comprise most of the biomass of even these relatively robust albino plants.

Albino growth tracks the seasonal activity of parent redwood trees. California’s coastal climate is characterized by wet winters followed by a generally dry growing period from April to September, during which frequent and heavy fog events provide an important source of hydration for coastal plants [5]. Hence, albino foliage is fully developed by late June and July, and typically dies back during the onset of the winter rainy season when assimilation in the parent trees is likely to decline.

Due to limitations on the number of available albino individuals, appropriately sized stems and sampling opportunities, samples are treated as statistically independent, similar to Lo Gullo et al. (2007) who encountered this problem in albino *Citrus*.

### Gas exchange and field measurements

Leaf chlorophyll content was measured using the CCM 300 chlorophyll meter (Opti-Sciences, Hudson, NH), using standard settings per Gitelson et al. [6].

To confirm that stems were devoid of photosynthetic activity, a Li-Cor 6400XT gas-exchange system (Li-Cor Biosciences, Lincoln, NE) was used to measure CO_2_ assimilation and dark respiration in both albino and green foliage. To ensure equivalent hydration, healthy and vigorous stem samples ranging from 3 to 6 mm in diameter and 20 to 25 cm in length were clipped in the morning, double-bagged with a wet paper towel, re-cut and trimmed in the lab, immersed in water, and left overnight to rehydrate in the greenhouse per Pittermann et al. [7]. Rehydration ensured that stem water potentials were between -1 and -0.5 MPa as measured by a pressure chamber (PMS Instruments, Corvallis, OR). The Li-Cor gas-exchange system was fitted with a standard 6 cm^2^ LED-lighted chamber, and leaf temperature ranged from 19–21°C, reference CO_2_ was set to 400 μmol mol^-1^, the flow rate was set to 300 ml/min, and vapour pressure deficit ranged from 1.1 to 1.25 kPa. Assimilation was measured at saturating light levels (light levels were raised incrementally to 1000 μmol m^-1^ s^-1^); respiration was measured with the chamber lights off. Under both light saturated and dark conditions, stability was reached after approximately 5–7 minutes. Measurements were taken mid-day between 10:30 am and 3:30 pm. The leaf area within the chamber boundaries was photographed and measured using ImageJ software [8] and used to recompute final assimilation/respiration rates.

On separate occasions in July 2011 (also January 2015), a porometer was used for hourly measures of stomatal conductance over the course of day (Decagon Devices, Pullman, WA); these data were coupled with measures of leaf water potential, temperature and ambient light levels. We did not have a humidity sensor available, so a range of vapour pressure deficit (VPD) levels was computed on the basis of air temperature, and known July relative humidity data which was collected in the redwood forest understory from 2012-present. Relative humidity varies from 45 to 65% in this habitat ([9–11]; R. Appleseed and J. Pittermann, unpub).

### Hydraulic measurements

Hydraulic measurements were conducted on stems used for gas-exchange as described above. After the gas-exchange measures, the stems were re-cut to approximately 160 mm under water, and xylem emboli were removed by degassing submerged stems overnight under vacuum in a filtered 20 mM KCl solution (<5 kPa suction; 0.22 μm; E-Pure filtration system; Barnstead International, Dubuque, IA). The stems were then trimmed to 142 mm, inserted in a tubing apparatus and hydraulic conductivity (*K*) was measured gravimetrically under a pressure head of 5 kPa using a filtered 20 mM KCl solution, while accounting for background flows [7,12]. Maximum conductivity (*K*_*max*_) was thus determined on fully rehydrated stems. Distal segments of these stems ranging from 4–7 cm in length were subsequently perfused with a basic fuchsin solution to identify the functional xylem tissue. Thin cross-sections of the stained xylem were mounted on glass slides, photographed using a Motic BA400 compound microscope (JH Technologies, San Jose, CA) and the stained areas measured using ImageJ software. Xylem specific conductivity (*K*_*s*_) and leaf specific conductivity (*K*_*leaf*_) were computed as *K*_*max*_ divided by the functional xylem area and distal stem leaf area, respectively.

Due to a handling mishap, the distal leaf area on a subset of stems was estimated from a regression of leaf area (mm^2^) as a function of stem diameter (mm) in green stems (*y* = 38539.4*x*—103873; R^2^ = 0.962). For a given stem diameter, albino leaf area is on average 49.6% lower than than of green redwoods (see also Table 1), so we adjusted the final albino distal leaf area accordingly. This is a conservative calculation based on leaf area:stem diameter ratios of vigorous young albino foliage, and thus overestimates the actual distal leaf area of the larger albino stems used for hydraulic measures. This is because albino twigs in nature tend to have fewer living leaves than similarly sized green twigs. Functionally, our reported average *K*_*leaf*_ values are conservative, and thus likely to be lower than for albino stems *in situ*. While not ideal, this leaf area correction was necessary due to the limited availability of appropriately sized material for hydraulic measures. We are confident that our approach correctly and realistically captures the *K*_*leaf*_ response of the stems in question.

**Table 1:** Anatomical, hydraulic and functional traits in green and albino redwood leaves.

Trait	Albino (mean ± SD; n)	Green (mean ± SD; n)	P-value (P<0.05)
**Chlorophyll Content (mg m-2)**	not detectable	763 ± 262; n = 5	NA
**Mean Stem Tracheid Diameter (**μ**m)**	13.2 ± 2.09; n = 11	13.8 ± 1.56; n = 10	NS
**Hydraulic Stem Tracheid** **Diameter (**μ**m)**	16.5 ± 2.26; n = 11	16.9 ± 1.82; n = 10	NS
**Stem Tracheid (t/D)h2**	0.1 ± 0.04; n = 7	0.18 ± 0.4; n = 7	0.015
**Leaf Mean Conduit Diameter (**μ**m)**	16.1 ± 1.89; n = 6	12.7 ± 1.96; n = 6	0.012
**Leaf Hydraulic Mean Conduit Diameter (**μ**m)**	17.8 ± 2.19; n = 6	14.7 ± 1.52; n = 6	0.0186
**Stem Specific Hydraulic Conductivity (kg m-1 MPa-1 s-1)**	0.8 ± 0.8; n = 7	0.9 ± 0.62; n = 6	NS
**Stem Leaf Specific Hydraulic Conductivity (kg m-1 MPa-1 s-1)**	1.4x10-4 ± 1.3 x 10–4; n = 7	6.2x10-5 ± 4.49x10-5; n = 6	NS
**Leaf Area:Xylem Area**	3628 ± 715; n = 6	5884 ± 609; n = 6	0.0002

Extended episodes of water deficit can generate significant tensions in the xylem sap, making conduits vulnerable to the entry and/or expansion of air bubbles. Expanding air may break the water column (cavitation), and subsequently create an air-vapour void (an embolus). Air-filled, embolized xylem cannot efficiently transport water, but plants have evolved varying degrees of cavitation resistance with respect to their habitat or life-history strategy; this can be compared within and between species [7, 9, 13]. The vulnerability of albino and redwood stems to embolism was evaluated in response to a range of xylem pressures using the centrifuge method [13, 14]. Stems were secured in a custom rotor designed for a Sorvall RC-5C centrifuge (Thermo-Fisher, Waltham, MA) and spun for four minutes at speeds known to induce a specific xylem tension (negative pressure; *P*_*x*_). Immediately after each spin, *K* was measured and used to compute the per cent loss of conductivity (PLC) relative to the maximum *K* obtained after degassing at a *P*_*x*_ of 0 MPa (*K*_*max*_), such that,

$$PLC=100x(1-\frac{K}{K_{\max}})$$ (1)

Stems were spun to progressively more negative *P*_*x*_ at 1 MPa increments until PLC exceeded 90%. The resulting ‘vulnerability curves’ were fit with a Weibull function [15] for each stem, and the fit was used to compute the *P50*, that is the *P*_*x*_ at which a stem loses 50% of its conductivity.

### Anatomical measures

Tracheid anatomy was evaluated on hand-made transverse sections collected from the middle portion of the centrifuged stems. The sections were stained with toluidene blue, mounted on a slide with a drop of glycerin, and photographed under 200-400X magnification. Three radial xylem sectors were photographed taking care to avoid reaction wood, and these corresponded to the most recently developed growth rings as confirmed by earlier perfusions with basic fuchsin. The lumen area of 60–90 tracheids was measured on each stem using ImageJ software [8]; lumen areas were subsequently converted to circle lumen diameters (*D*; [7, 16]). The hydraulically weighted conduit diameter was computed for each stem as ∑(*D*^5^)/∑*D*^4^) per Kolb and Sperry [17]. A similar approach was used to measure leaf xylem conduit diameter, except that the xylem tissue was sampled from the base of the phyllode; lumen area was measured on 40–70 tracheids per stem.

Sap tension in the xylem imposes hoop and bending stresses on conduit cell walls, making cells vulnerable to collapse unless they are sufficiently fortified to resist implosion [16, 18, 19]. Since large-diameter conduits contribute disproportionately more to sap flow than narrow ones, conduit reinforcement was quantified on cells that are within ± 10% of the mean hydraulic conduit diameter by measuring the cell double-wall thickness (*t*) of two walls, which was then averaged and used to compute (*t/D*)_h_^2^ (n = 15 to 30 cells; [18]).

The thickness of the three most recently formed growth rings was measured in the three sectors of the above-mentioned xylem thin sections. Stems were photographed under 100–200X magnification, and the growth ring thickness was measured using ImageJ software.

Stomatal density (# stomata/mm^2^) was counted on both the abaxial and adaxial sides of the leaves. A thin layer of clear nail polish was painted on the surface of the youngest, most recently expanded leaves, gently peeled off with tweezers, and mounted on slides with glycerin. One leaf was sampled from the centre of each phyllode. Stomatal density was counted within the proximal, central and distal portions of both sides of the leaf under 100–200X magnification.

### Statistical analysis

All statistical analyses were performed using the standard R software package [20]. Although *K* and *K*_*leaf*_ distributions were not normally distributed due to identifiable outliers, these data were within the norm of published values as well as the expected variation in the data set, and were thus retained in the analysis. Variance was similar between the green and albino response, so a Welch’s T-Test was used to compare between treatment (albino) and control (green) results.

## Results

Albino redwoods had no detectable levels of chlorophyll in comparison with adjacent green foliage, in which chlorophyll content ranged from 486 to 2094 mg m^-2^ (Table 1).

**Fig 2:**
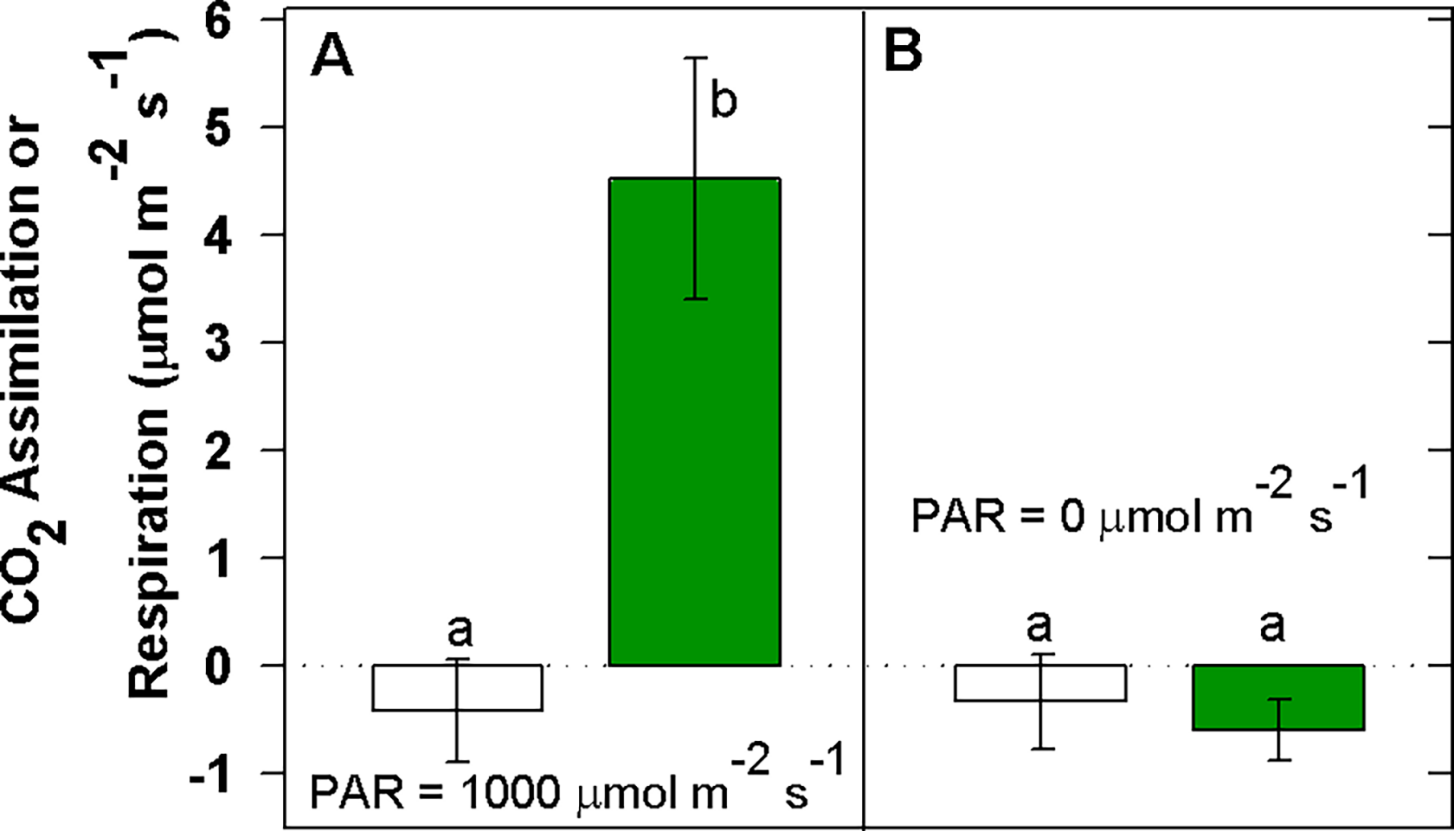
Photosynthesis and respiration in albino and green foliage. Gas exchange was measured under saturating light (A; n = 10 albino leaves, n = 6 green leaves) and in the dark (B; n = 13 albino leaves; n = 8 green leaves).

Consistent with this, exposure of green leaves to saturating light stimulated photosynthesis to 4.5 ± 1.12 μmol m^-2^ s^-1^ (mean ± SD), which is typical for understory redwood foliage, but no effect was observed in albino leaves which respired at an average rate of -0.4 ± 0.48 μmol m^-2^ s^-1^ (Fig 2).

Dark respiration was not significantly different between the albino and green leaves.

**Fig 3:**
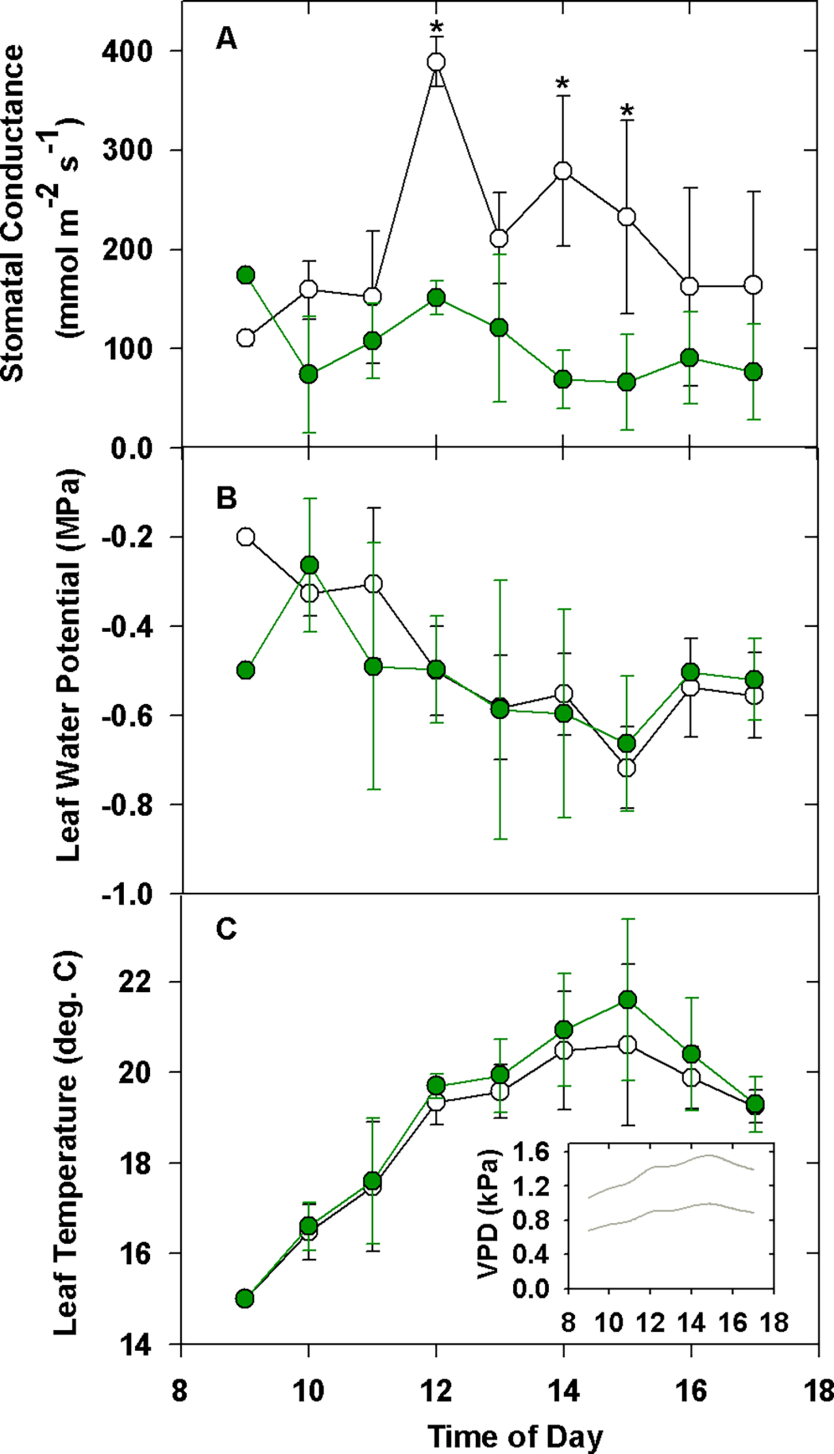
Field measures of leaf water loss in albino and green redwood leaves. Stomatal conductance (A), leaf water potential (B) and leaf temperature and vapour pressure deficit (C, inset) on albino and green redwood foliage *in situ* (n = 4 individual albino shrubs; n = 3 individual green shrubs; mean ± SD). Asterisk indicates significant differences (P < 0.05) between green and albino responses.

Over the course of a day, stomatal conductance *in situ* was almost always two to three times higher in albino leaves than in green redwoods, reaching a maximum of 388 ± 25.3 mmol m^-2^ s^-1^ at 12:00 (Fig 3A).

The largest differences at mid-day are presumed to be in response to increasing vapour pressure deficit (Fig 3A and 3C). By contrast, no difference was observed between the albino and green foliage with respect to hydration status: leaf water potential ranged from -0.2 to -0.7 MPa in both types over the course of the day (Fig 3B). Similar patterns were seen in January (S1 Fig; Henry Cowell State Park individuals only). Leaf temperatures in albino and green foliage are statistically indistinguishable.

**Fig 4:**
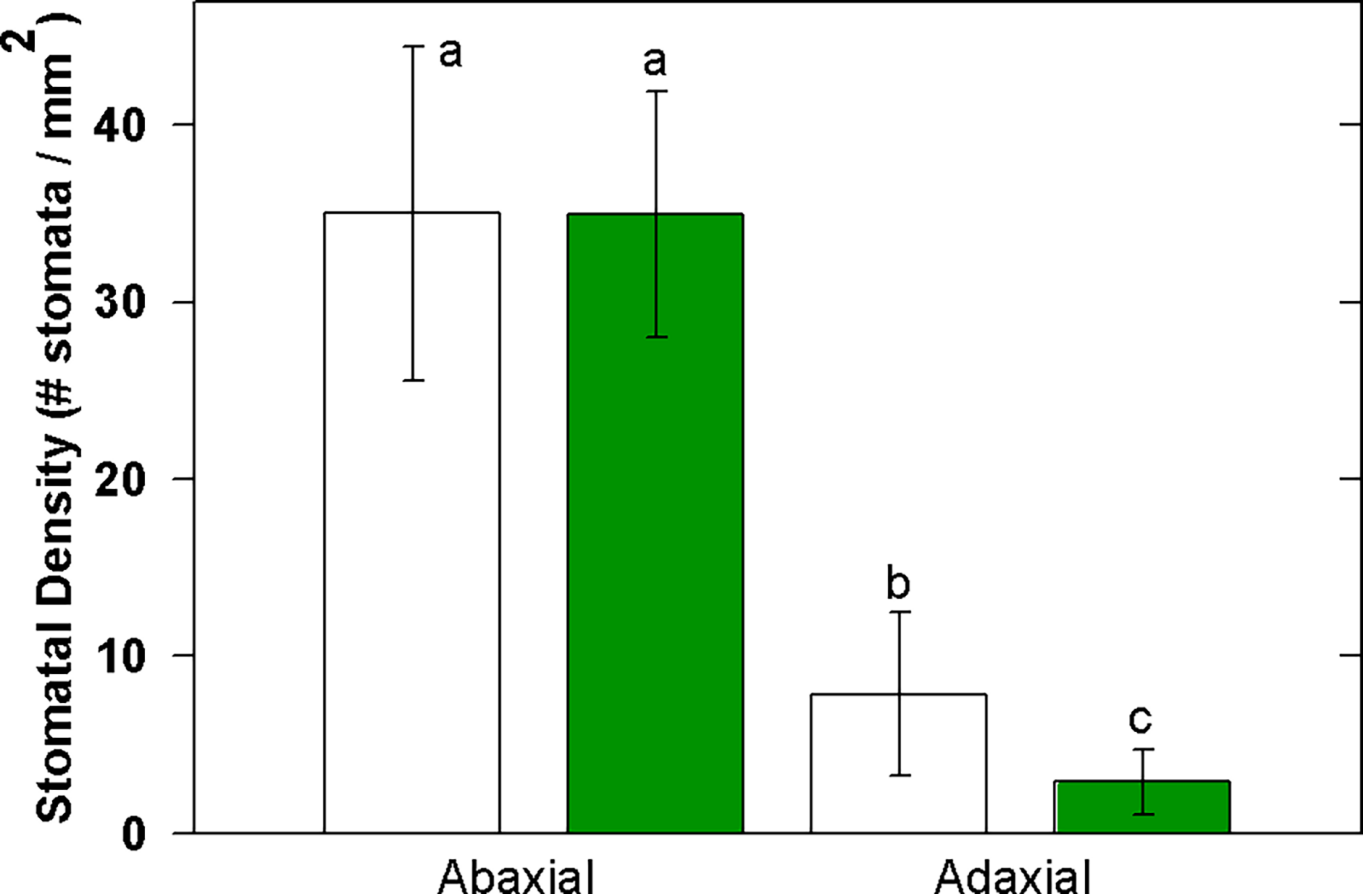
Stomatal density on albino and green leaves on both abaxial and adaxial sides.

Stomatal density on the abaxial side of the leaf was nearly equivalent for both albino and green redwoods, averaging 35 and 34.86 stomata mm^-2^ respectively (Fig 4; n = 11 albino leaves, n = 10 green leaves). However, albino leaves had significantly greater numbers of stomata on the adaxial side (7.9 ± 4.58 stomata mm^-2^; P = 0.013, mean ± SD) than green leaves (2.9 ±1.84 stomata mm^-2^).

**Fig 5:**
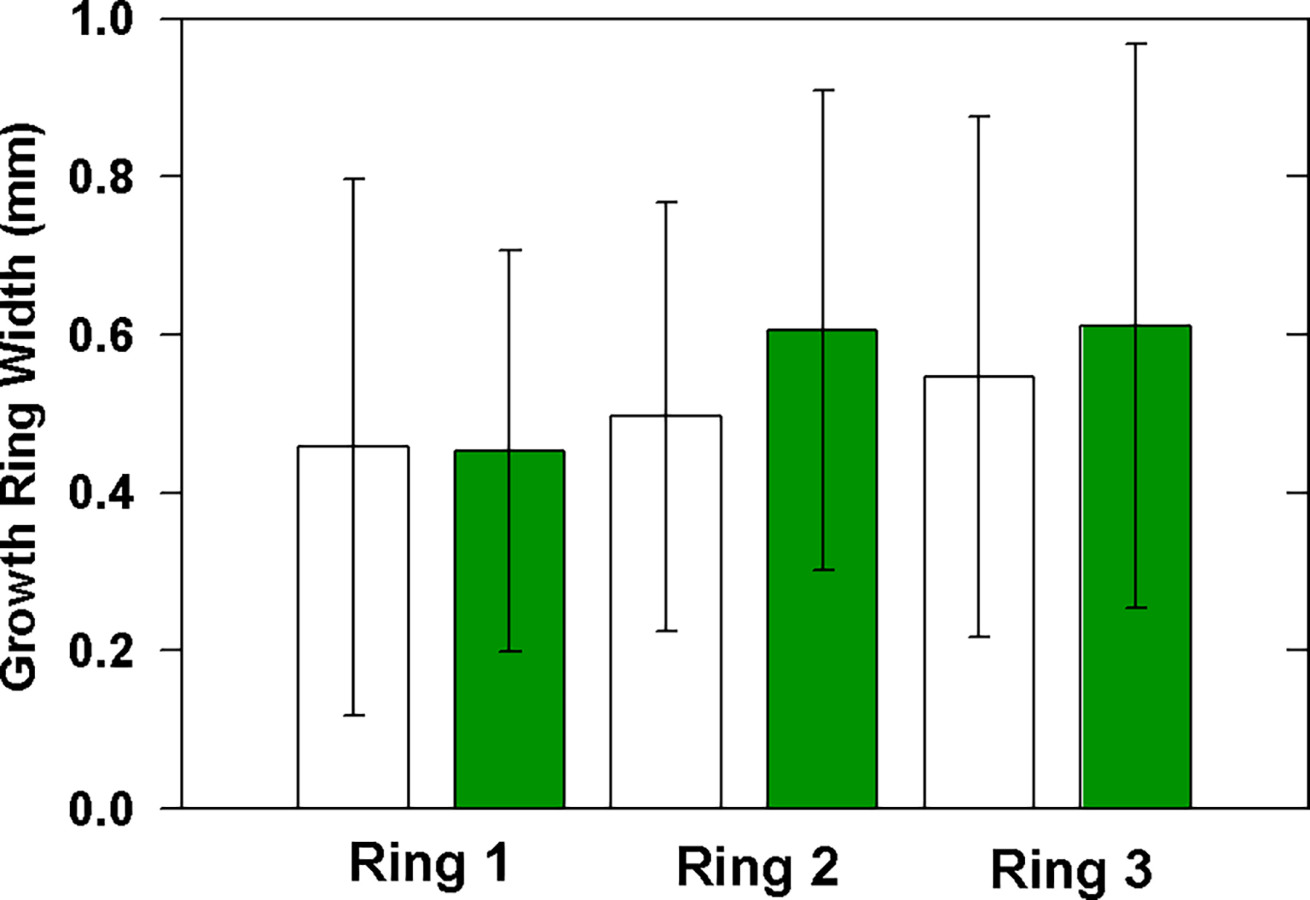
Annual growth rings in stems of albino and green redwoods.

Growth ring widths were similar in albino and green stems (Fig 5; n = 11 albino, n = 10 green stems). Samples were taken in July so the most recent growth ring may not be fully developed. We expected that albino sprouts attached to photosynthesizing trees would have larger growth rings than those growing from stumps, but found no association between albino growth rings and perceived tree vigor. Indeed, the growth of albino sprouts is seemingly sporadic even on living trees, suggesting that factors other than the metabolic activity of the parent plant may be at play.

Stem xylem-specific hydraulic conductivity was nearly equivalent in albino and green stems respectively (Table 1). This is consistent with a similar mean tracheid diameter as well as the hydraulic mean conduit diameter in both types of stems ~13 and 16 μm; Table 1). By contrast, the hydraulic and mean tracheid diameters in the phyllode mid-rib were 17–20% wider in albinos than in the green foliage (Table 1).

Average leaf-specific conductivity (*K*_*leaf*_) in albino sprouts was over twice as high as in the green samples, although the variation within treatments rendered the differences statistically insignificant (P = 0.18; Table 1). The data are however, consistent with the significantly lower leaf area to xylem area ratios in albino stems relative to green stems (Table 1). Put another way, albino sprouts have on average 38% lower leaf area for a given amount of xylem than green sprouts, presumably because the absence of photosynthesis limits albino leaf growth rates. More broadly, these data in combination with the slightly larger tracheids in albino leaves, are consistent with the albino leaves’ higher rates of water loss (Fig 3A).

**Fig 6:**
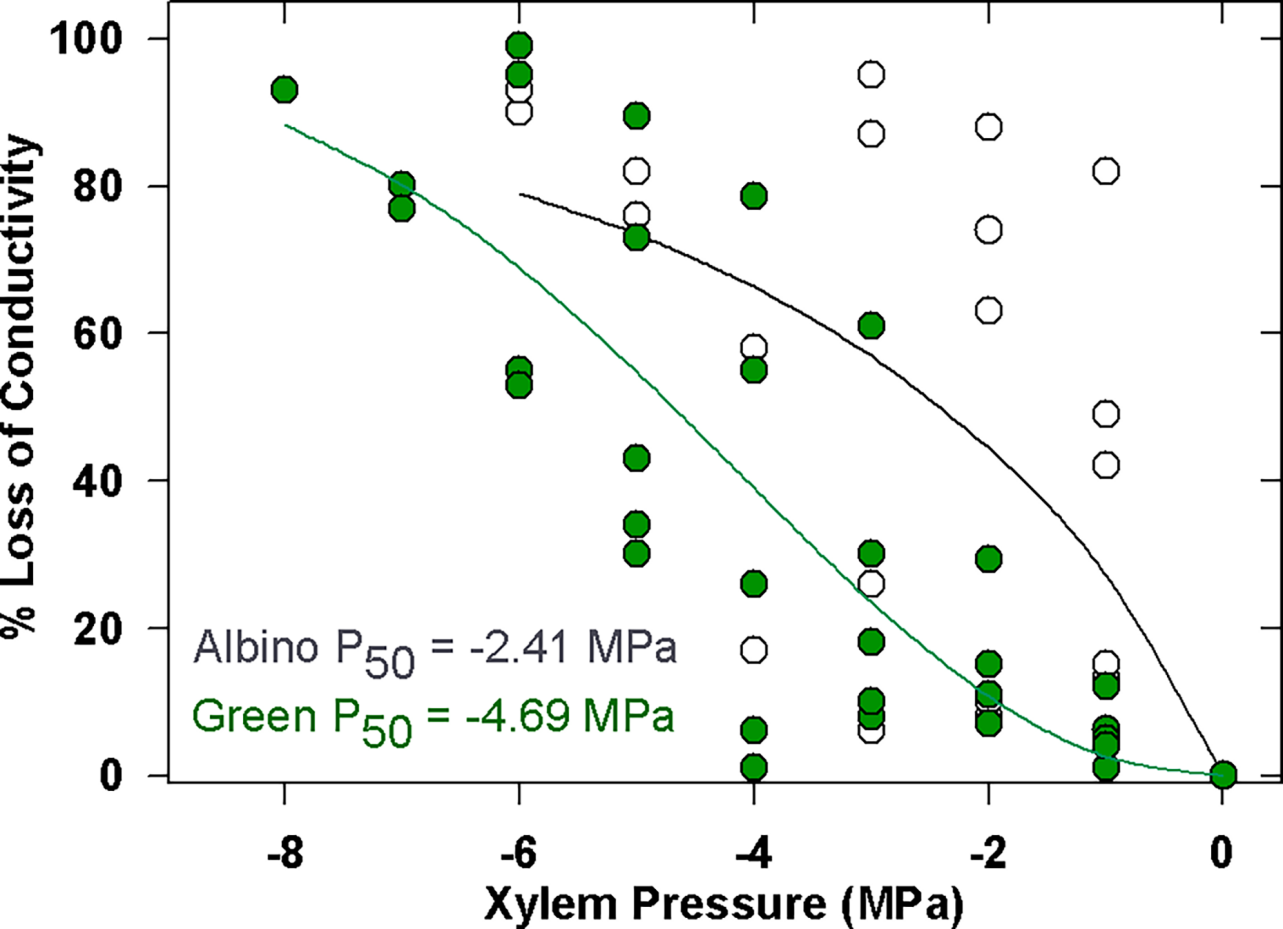
The percent loss of conductivity in albino and green redwood twigs in response to increasing xylem sap tension. A combined curve fit is shown for the green and albino material, although stems were fit individually for statistical analysis.

We predicted that albino xylem would be weaker and more vulnerable to cavitation, and this was indeed the case (Fig 6; n = 5 albino, n = 5 green; P = 0.032).

By contrast, the green stems generally exhibited a much greater resistance to cavitation, with an average P50 of -4.7 ± 1.39 MPa in contrast to the albino stems, in which P50 averaged -2.5 ± 1.37 MPa. The albino stems were brittle and thus more susceptible to cracking and breakage during centrifugation; only two stems were robust enough to tolerate -6 MPa in the centrifuge. The significantly lower (*t/D*)_h_^2^ ratios in albino xylem are in line with the generally higher vulnerability of these stems to cavitation, and indicate that the albino xylem is structurally weaker (Table 1).

All data are available in the manuscript supplement (S1 File).

## Discussion

The goal of this study was to examine the eco-physiological and anatomical consequences of naturally occurring albinism in *Sequoia sempervirens* foliage. Studies of holoparasites and albino angiosperms have shown that the absence of chloroplasts has a profound effect on stomatal conductance, leaf structure and xylem function [3, 4, 21, 22], but to our knowledge, our research is the first to examine these traits in an albino conifer. Overall, there appears to be a general convergence of plant functional responses to albinism because the lack of chlorophyll imposes limits on carbon gain, development and stomatal function. As in achlorophyllous angiosperms, albino redwood mutants exhibit much higher rates of leaf water loss than green foliage, and a total absence of photosynthetic carbon gain in spite of respiration rates that are comparable to green leaves [3, 23]. These physiological responses translate to interesting shifts in upstream vascular function. Specifically, reduced carbon gain results in less leaf area and thus lower leaf to xylem ratios, and this is consistent with higher stomatal conductance in albino sprouts relative to those that are green (Table 1). Furthermore, albino xylem is structurally weaker and more susceptible to drought-induced embolism. Taken together, unlike parasitic plants which evolved to extract carbon, water and nutrients from host plants for the purpose of enhanced fitness, albino sprouts are simply a resource sink, much like the albino foliage of *Citrus* and *Nerium* [3, 4]. Yet despite their random occurrence, albino redwoods do offer a valuable opportunity to explore alternative plant function in the absence of a critical trait.

Leaf water loss was generally higher in albino mutants than in green leaves, although the total amount of water lost per shoot was probably similar due to shifts in distal leaf area. Albinos almost always had higher stomatal conductance than their green counterparts, whether in the summer or winter (Fig 3; S1 Fig), and in the absence of a response to light or decreasing water potentials, water loss from albino foliage appeared to be unregulated. Similar patterns were found in achlorophyllous angiosperms. For example, both albino *Citrus* and *Nerium* spp. had rates of leaf water loss that were 1.5× higher than those of green shoots; this response was especially pronounced under warm conditions when VPD exceeded 1.5 kPa (see also reference [3]). Leaf stomatal density was also much higher in *Citrus* [3]. Much less is known about gymnosperms in this regard, but the understory conifer *Parasitaxus ustus* was also found to have greater stomatal conductance than its host, and a reduction in stomatal conductance occurred concurrently with dropping water potentials [24]. The response in the albino redwoods was less clear. Water potential, which was related to VPD and leaf temperature, declined from -0.3 to -0.7 MPa over the course of the day in both green and albino foliage, but in contrast to *Citrus*, stomatal conductance was not coupled to VPD in a straightforward manner (Fig 3). Site and plant heterogeneity may explain the absence of a relationship, as well as the narrow range of temperatures and VPD values under which the data was collected; the full extent of the potential stomatal response may not be revealed under mild conditions. In contrast to the *Citrus* orchard where ambient temperatures reached a maximum of 28.7°C [3], temperatures in the redwood forest understory did not exceed 22.6°C.

High rates of water loss in albino leaves may be intertwined with other aspects of redwood leaf function. For example, redwoods may not have evolved strong stomatal regulation or they are simply incapable of it, as they lose a considerable amount of water nocturnally [25]. This may be amplified by redwood genetics because *S*. *sempervirens* is a hexaploid species: stomatal size increases with ploidy level [26, 27] but by the same turn, stomatal dynamics become less efficient with increasing size [28] so *Sequoia's* polyploidy may reduce stomatal efficiency or hinder complete sealing by the guard cells. Furthermore, the absence of chloroplasts in the guard cells undermines critical light-dependent signaling pathways that would otherwise stimulate closure, with the effect of leaving the guard cells turgid and the stomata open ([29, 30] and references therein). Stomata also respond to leaf internal CO_2_ levels [31, 32] so in the absence of photosynthesis, this cue is unlikely to exist. Complicating this trifecta of problems is a weakly developed epidermal layer and cuticle in the albino leaves (Fig 1C and 1D), so in contrast to green foliage, albino leaf water loss may also be through the dermal tissue. Neither a porometer nor a gas-exchange system can distinguish between cuticular or stomatal water flux.

The effects of higher leaf water loss on albino xylem structure were subtle. Lo Gullo et al. [3] show that the higher transpiration rates in albino *Citrus* twigs are supported by significantly larger xylem vessels, and other studies linking leaf water loss with conduit diameter find a similar degree of vascular plasticity [33]. Somewhat surprisingly, stem tracheid diameters were nearly identical in both albino and green redwood sprouts. Significantly larger tracheids were observed in albino leaves (phyllodes; Table 1), but this finding alone is insufficient to account for the large disparity between water loss in albino and green plants. Ultimately, slower leaf growth rates and fewer leaves per twig favour the water relations of albino sprouts: low leaf area to xylem area ratios and by extension, high leaf-specific conductivity help explain the high stomatal conductance in albinos. With its highly reduced foliage, *Parasitaxus* employs a similar strategy [24], but in contrast to this specialized parasite, elevated stomatal conductance in albino sprouts results from impaired leaf function, rather than being a *bona fide* adaptive trait.

The albino's reduced carbon gain had a profound effect on xylem quality. Growth rings in albino twigs were narrower than those of green sprouts, indicating slower growth rates, although the developmental program of the cambium was not significantly disrupted with respect to tracheid size and growth ring width. The cost however, was the production of structurally weak tracheids, as indicated by the albino's lower (*t/D*)_h_^2^ values (Table 1). Weak conduits align with the albino's increased vulnerability to cavitation [7, 16, 18], but future work should examine the role of the pit membrane because delicate microfibrils and imperfectly developed tori are known to affect cavitation resistance [34–36]. Indeed, the functionality of albino xylem has the semblance of *Taxodium distichum*, a hydric species with low-density xylem that is vulnerable to embolism due to thin pit membranes [34, 35]. However, an important difference is that unlike in *T*. *distichum*, vulnerable xylem in albino redwoods is not compensated by increased hydraulic efficiency, which results from larger tracheids and more conductive pit membranes [7, 16, 35]. Indeed, albino xylem falls in the 'vulnerable yet hydraulically' inefficient trait space recently described by Gleason et al. [37].

## Conclusion

The height, girth and intrinsic beauty of redwood trees can be attributed, in large part, to their unusually expansive growth, which is a function of their vascular cambium, among other developmental, physiological and life history attributes. While the specific lesion that leads to redwood albinism has yet to be identified, it could arise during cell division in the cambial layer or the development of buds from burls. In any case, a reasonable explanation is that plastids fail to divide (or divide unevenly) during mitosis, leaving one daughter cell incapable of developing chloroplasts. Whatever the cause may be, the absence of chloroplasts in albino twigs impedes stomatal function, while the inability of the leaves to photosynthesize markedly diminishes xylem quality, and reduces the growth rates of both leaves and xylem. Although seed cones were said to be observed on albino sprouts by Santa Cruz locals, the likelihood of any albino seed generating a viable plant is close to nil. Yet much can be learned from naturally occurring gene knockouts and without a doubt, leaf albinism can be added to the list of traits that make redwoods such charismatic trees.

## Supporting information

S1 Fig Stomatal conductance (A), leaf water potential (B) and leaf temperature on albino and green redwood foliage *in situ* measured in January 2015. (n = 2 individual albino plants; n = 2 individual green plants).
(TIF)

S1 File Raw data used to compute the means and standard deviations of the physiological and anatomical attributes reported in the manuscript. (CSV)
